# Pentamidine-Functionalized Polycaprolactone Nanofibers Produced by Solution Blow Spinning for Controlled Release in Cutaneous Leishmaniasis Treatment

**DOI:** 10.3390/polym18020170

**Published:** 2026-01-08

**Authors:** Nerea Guembe-Michel, Paul Nguewa, Gustavo González-Gaitano

**Affiliations:** 1 Department of Chemistry, School of Science, University of Navarra, 31080 Pamplona, Spain; nguembe.1@alumni.unav.es; 2 Department of Microbiology and Parasitology, Navarra Institute for Health Research (IdisNA), University of Navarra, 31080 Pamplona, Spain

**Keywords:** solution blow spinning, polycaprolactone, nanofibers, drug kinetics, pentamidine, leishmaniasis

## Abstract

Leishmaniasis, a widespread, neglected infectious disease with limited effective treatments and increasing drug resistance, demands innovative therapeutic approaches. In this study, we report the fabrication of pentamidine (PTM)-loaded polycaprolactone (PCL) nanofibers using solution blow spinning (SBS) as a potential topical delivery system for cutaneous leishmaniasis (CL). Homogeneous PCL fiber mats were produced using a simple SBS set-up with a commercial airbrush after optimizing several working parameters. Drug release studies demonstrated sustained PTM release profile over time, which was mechanistically modeled by utilizing the complete nanofiber diameter distribution, obtained from SEM analysis of the blow-spun material. FTIR and XRD analyses were performed to investigate the drug–polymer interactions, revealing molecularly dispersed PTM at low-proportion drug/polymers and partial crystallinity at high loadings. The released PTM exhibited significant leishmanicidal activity against *Leishmania major* promastigotes. Biological investigations showed that SBS-formulated PTM treatment was consistent with the downregulation of parasite genes involved in cell division and DNA replication (*cycA*, *cyc6*, *pcna*, *top2*, *mcm4*) and upregulation of the drug response gene (*prp1*). The controlled delivery of PTM within SBS-fabricated PCL nanofibers provides an effective therapeutic approach to tackle CL and, through the incorporation of additional drugs, could be extended to address a broader range of cutaneous infections.

## 1. Introduction

Leishmaniasis, a neglected tropical disease (NTD) as classified by the World Health Organization (WHO), is a vector-borne disease caused by protozoan parasites of the genus *Leishmania* [1] that are transmitted by female sand flies of *Phlebotomus* and *Lutzomyia* spp. The parasite’s digenetic life cycle involves promastigote (flagellated and elongated form) proliferation in the insect vector and amastigote (spherical intracellular form) replication within mammalian macrophages and endothelial cells [2,3,4,5,6,7].

Leishmaniasis can be caused by various species of *Leishmania*, mainly *L. major*, *L. infantum*, *L. amazonensis*, *L. donovani*, *L. tropica*, and *L. braziliensis* [2]. These are responsible for different clinical manifestations of the disease, such as visceral (VL), mucocutaneous (MCL), and cutaneous (CL) leishmaniasis. The latter is the most common in humans and occurs in the form of ulcers on the skin, sometimes accompanied by swollen glands. Currently, the distribution of vectors and parasites is evolving due to climate change, threatening new regions where leishmaniasis has never been a problem, such as northern Europe or southern areas of South America [8,9,10].

Present treatments against leishmaniasis include amphotericin B [11,12], miltefosine [5,6,13,14], pentavalent antimonials [11,15], paromomycin [16,17,18], and pentamidine [19]. Pentamidine (PTM) is an antifungal drug with demonstrated antileishmanial activity, which is thought to involve interference with polyamine synthesis, inhibition of RNA polymerases, and binding to parasite DNA and proteins [20]. However, PTM therapy is limited by adverse effects such as hypotension, cardiac alterations, hyperkalemia, hypoglycemia, severe myalgias, and hepatotoxicity, as well as by the development of parasite resistance via reduced drug influx and enhanced efflux through the PRP1 transporter [19,20]. As these medications are often associated with toxicity, high costs, and increasing drug resistance, it is necessary to explore novel therapeutic strategies and formulations, including immunomodulators [21], vaccines [8,22], antimicrobial peptides [23,24], selenium derivatives [25,26], liposomes [27,28], nanoparticles [27,28,29,30,31], or polymeric fibers.

In this context, biomaterials based on biocompatible polymers for topical delivery can offer benefits for improving the treatment of CL by enhancing drug concentration and permeation on the skin lesions while minimizing systemic side effects. Among various biomaterials-based platforms, polymeric micro- and nanofibers are gaining interest due to their ability to provide a high surface area for drug loading and controlled release, promoting localized and sustained therapeutic action [31,32,33,34,35], as well as inducing cell proliferation [32,33]. Additionally, micro- and nanofibers can be tailored to incorporate other molecules along the antiparasitic drugs, such as antifungals, antibiotics, anti-inflammatory drugs, and other therapeutic agents [33,34,35].

The most commonly used methods of micro- or nanofiber production are electrospinning and Solution Blow Spinning (SBS) [33,36]. Electrospinning (ES) stands as a widely employed technique for fiber production. This method involves pumping a polymer solution through a syringe at a controlled rate under a high voltage, which overcomes the solution’s surface tension and results in fiber formation [36,37]. ES offers versatility in material selection, cost-effectiveness, and the possibility of fiber functionalization before or after the process, although has limitations in terms of the scalability for mass production [32]. SBS [38], has emerged as a compelling alternative to traditional ES, as it offers the advantage of the in situ fiber generation [39]. This technique utilizes the gas flow from a compressor to generate fibers from a polymer solution, offering a much simpler set-up compared to ES [36,39,40] and the possibility of a fast elimination of poorly eco-friendly solvents compared to ES.

The selection of the polymer for biomedical applications is based on its biocompatibility, degradability, and fiber-forming ability [36,41]. Commonly used materials include polyvinylpyrrolidone (PVP) for its hydrophilicity and smooth fiber formation, poly(lactic acid) (PLA) and polycaprolactone (PCL) for their biodegradability and mechanical properties, and polyvinyl alcohol (PVA), polyvinyl chloride (PVC), and chitosan, which provide additional functionalities such as solubility, structural support, or enhanced cell adhesion [6,36,41,42,43]. These characteristics directly affect fiber morphology, mechanical behavior, and therapeutic performance, allowing for customized designs tailored to specific biomedical needs. Recently, the potential of SBS to address leishmaniasis was demonstrated with formulations of hydroxypropyl-β-cyclodextrin and miltefosine embedded in nanofibrous mats of PVP and the amphiphilic block copolymer Tetronic^®^ 1307 [6]. Other studies of SBS include the investigation of nanofiber mats for skin wound dressing applications, such as the encapsulation of curcumin in PCL fibers [44], and ibuprofen or iodine in PCL/polyethylene glycol (PEG) fiber mats [45,46]. Likewise, electrospun-produced mats have been explored for leishmaniasis treatment by encapsulating sorafenib in PCL/cellulose acetate mats [47], as well as glucantime and quercetin in PCL mats [48]. However, to our knowledge, there are no studies reporting on the formulation of drugs in PCL–fiber matrices for leishmaniasis treatment using SBS, which would permit the in situ deposition of the material on *Leishmania*-caused wounds.

In this work, we hypothesize that PTM-loaded blow-spun fibers could enable sustained and localized drug release suitable for topical treatment of CL. To this end, for the first time, we developed a sprayable PTM-based formulation of nanometer-sized PCL fibers produced by SBS, an approach that allows for direct, on-demand deposition of functionalized mats on the cutaneous lesions produced by CL. The experimental set-up, based on a handheld airbrush coupled to a rotating drum collector, was optimized to produce highly homogeneous and reproducible fibrous mats whose morphology, structural properties, and chemical integrity were fully characterized by Scanning Electron Microscopy (SEM), Fourier-Transformed Infrared (FTIR) spectroscopy, and X-ray diffraction (XRD). The sustained release behavior of PTM from the fibers was justified by using a kinetic model that integrates the whole fiber diameter distribution, providing a mechanistic description of the release process. The antileishmanial activity and cytotoxicity of the released PTM was then assessed in vitro against *Leishmania* promastigotes and on macrophages, respectively. Finally, the potential mechanism of action of PTM was explored, evaluating the consistence of the treatment with the expression levels of several leishmanial genes related to cell cycle and division, treatment resistance, and DNA replication.

## 2. Materials and Methods

### 2.1. Materials

Polycaprolactone (PCL, 80,000 g/mol, CAS 24980-41-4) and dichloromethane (DCM, CAS 75-09-2) were purchased from Sigma-Aldrich (Darmstadt, Germany). Pentamidine isethionate (PTM, CAS 140-64-7) was purchased from Glentham Life Sciences (Corsham, UK).

### 2.2. Fiber Production and Characterization

#### 2.2.1. Fiber Production via SBS

**Figure 1:**
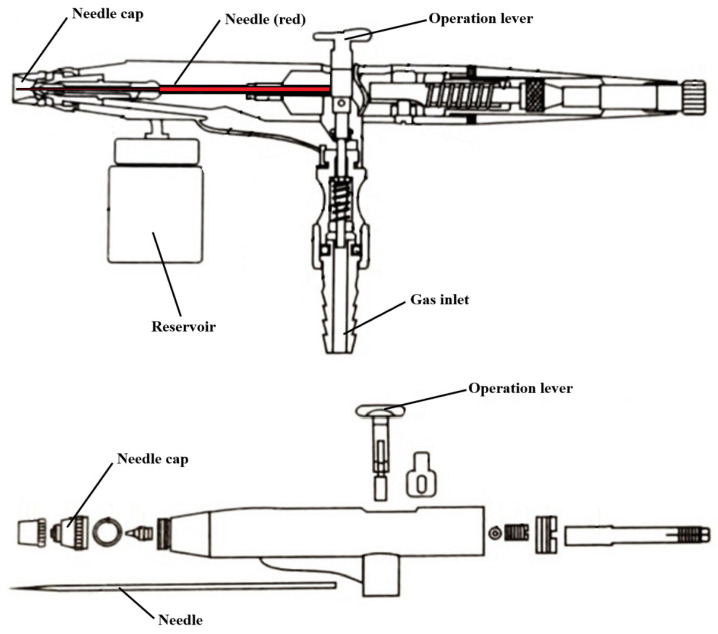
Schematic diagram of the commercial airbrush and its components, which enable the extrusion and controlled fiber formation.

The experimental set-up consisted of a commercial airbrush siphon feed (CYBERNOVA SP134T) (Figure S1). A schematic representation of the airbrush and its components is shown in Figure 1. The commercial airbrush operates by allowing for compressed air to flow through the body of the device, creating a pressure drop at the nozzle that draws the polymer solution from the reservoir. The polymer solution is then extruded in the nozzle, aided by the position and motion of the internal needle and the gas flow (Video S1). Both needles share the same shaft diameter; however, they differ in the diameter of the needle tip, either 0.3 mm or 0.5 mm (Figure S2). A solution of PCL in DCM, with concentrations ranging from 4% to 8%, was introduced into the reservoir and airbrushed by passing an air current from a compressor (between 2 and 3 bar). As the solvent evaporates during the flight time, the nanofibers (Figure S3) are deposited on a cylindrical spinning collector made of metallic mesh rotating at speeds between 150 and 350 rpm. The distance between the nozzle and the collector was fixed at either 15 cm or 20 cm.

#### 2.2.2. Morphology of the Fibers

The texture and general features of the resulting material were first characterized by polarized light microscopy using an Axiolab reflection microscope (Zeiss, Oberkochen, Germany) on fibers projected directly on glass slides. SEM was then used for the analysis of the fiber width distribution, after the optimization of the experimental set-up established by the optical microscopy observations. The samples were projected on cylindrical sample holders, gold-coated (EMITECH K550), and then inspected using a scanning electron microscope (Zeiss Sigma 300 VP) in secondary electron (SE) mode. Image analysis was carried out with the Ridge Detection plugin implemented in FiJi software [49], analyzing six micrographs of each set of SBS set-up conditions. Fiber diameter measurements were obtained from 65,535 data points for the 0.3 mm needle tip and 65,536 data points for the 0.5 mm needle tip. Distributions were calculated from SEM images using OriginPro 8.5 [50] with 113 bins for both needle sizes. The distributions were bimodal: for the 0.5 mm needle, the two modes contained 16,228 and 49,308 measurements, respectively, and for the 0.3 mm needle, the modes contained 37,749 and 27,786 measurements, respectively.

#### 2.2.3. Determination of the Surface/Weight Ratio and Thickness of the Mats

Cut-outs of the SBS-produced mats (polymer-only samples and polymer–drug formulations) were weighed and their dimensions measured with a digital caliper. To calculate the surface/weight ratio, the thickness of the mats was measured at the center and at the edges (Figure S4) of the same samples used for the surface/weight analysis.

#### 2.2.4. Drug Loading Determination

Specimens of the PCL/PTM bandages used for the surface/weight and thickness analyses were dissolved in 10 mL of DCM. The drug was then extracted via liquid–liquid extraction using three sequential 4 mL aliquots of distilled water due to the lack of solubility of the drug in DCM. A PCL mat and a 1 mM PTM standard were processed the same way to monitor background fluorescence and to determine the extraction yield, respectively. The fluorescence of the aqueous extracts was measured to obtain the actual PTM proportion in the bandages, from which the drug loading and encapsulation efficiency were determined. An Edinburgh Instruments (Livingston, UK) FLS920 spectrofluorimeter was used to this effect after calibration with PTM standard solutions, measuring the fluorescence of the extracts in 1 cm path-length quartz cells, with excitation and emission wavelengths of 270 and 340 nm, respectively.

#### 2.2.5. FTIR-ATR Spectroscopy

FTIR spectra were recorded from 600 to 4000 cm^−1^ using a Shimadzu IRAffinity-1S spectrophotometer (Kyoto, Japan) fitted with an Attenuated Total Reflectance (ATR) accessory (Golden-Gate, Specac) with a diamond window. For each sample, 32 scans were acquired, applying Happ-Genzel apodization to perform Fast Fourier Transform (FFT) of the averaged interferogram. To enhance statistical accuracy, the spectral resolution was increased in the experiment from 4 cm^−1^ to 1 cm^−1^ using polynomial interpolation with OMNIC 6.0 software [51].

#### 2.2.6. X-Ray Diffraction (XRD)

PTM powder and bandages of PCL and PCL + PTM were characterized by XRD at room temperature in a Bruker (Billerica, MA, USA) D2 Phaser diffractometer employing the Cu K_α1_ radiation (1.5406 Å) source applied at 30 kV and 10 mA. Diffraction patterns were recorded over a 2θ angle range from 5° to 70° using a step size of 0.02° (2θ) and a counting time of 2.5 s per step. The diffractograms were background-corrected using the implemented DIFFRAC.EVA software [52].

### 2.3. Drug Release Kinetics

PTM functionalized fibers were produced by incorporating the drug into the PCL/DCM solution. For a given polymer/solvent ratio, the corresponding amount of PTM was added, and the resulting mixture was used to feed the airbrush. Ca. 130 mg of collected fibers were weighed and cut into rectangular homogeneous pieces for their utilization in the drug release tests. The mats were placed in 600 mL of distilled water at 25 °C and sampled at different times for 5 days (120 h) using a Sotax AT 7 Smart Semi-Automated Dissolution Tester. The concentration of released PTM was quantified by fluorescence spectroscopy, as explained in Section 2.2.4. The experimental data were then fitted using a custom model developed in MATLAB R2024b, as described in the Supporting Information (Script S1).

### 2.4. Culture Conditions

*Leishmania major*, *Leishmania infantum*, and *Leishmania amazonensis* promastigotes were cultured at 26 °C in M199 1× medium supplemented with 0.1 mM adenine, 0.0005% (*w*/*v*) hemin, 25 mM HEPES (pH 7.4), 1 mg/mL biopterin, 0.0001% (*w*/*v*) biotin, 10% (*v*/*v*) heat-inactivated fetal bovine serum (FBS), and an antibiotic cocktail (50 U/mL penicillin and 50 mg/mL streptomycin) [5,14,26,53]. RAW264.7 macrophages were cultured at 37 °C and 5% CO_2_ in Dulbecco’s Modified Eagle Medium (DMEM) supplemented with 10% (*v*/*v*) heat-inactivated Fetal Bovine Serum (FBS) and 2% penicillin/streptomycin.

### 2.5. Leishmanicidal Activity in Promastigotes

The activity of PTM against *Leishmania* promastigotes was studied by the MTT method. 3-(4,5-dimethylthiazol-2-yl)-2,5-diphenyltetrazolium bromide reagent (MTT) was diluted in PBS at 5 mg/mL and kept at −20 °C [5,14,26,53]. In this colorimetric assay, several concentrations (0–10 µM) of free PTM, as well as different concentrations (0–100%) of nanofiber-released PTM, were tested in order to evaluate the leishmanicidal effect of the drug in promastigotes.

A Neubauer chamber was used to count parasites. After a 48 h incubation period, 20 µL of MTT were pipetted into each well so that the parasites were able to metabolize the reagent for 4 h. Then, 80 µL of dimethyl sulfoxide (DMSO) were added to dissolve the crystals formed during the metabolization of MTT. The absorbances of the colored solutions were measured at 540 nm using a Multiskan SkyHigh spectrophotometer. The resulting absorbance data were then used for the determination of the IC_50_ (inhibitory concentration 50) values, which were calculated with OriginPro 8.5 software [50] with the Boltzmann equation (Equation (S1)). MTT assays were performed with four technical replicates per treatment concentration (eight for controls) in each experiment. Data from each experiment were averaged, and the final results represent the mean ± SD (standard deviation) of all experiments. Randomization was applied for sample allocation, but measurements were not blinded.

### 2.6. Cytotoxicity on Macrophages

The cytotoxicity of PTM was assessed using RAW 264.7 macrophages, which were seeded (2 × 10^4^ per well) in 96-well plates. The cells were then treated with increasing concentrations of the drug for 48 h. Macrophages were counted using a Neubauer chamber. After a 48 h incubation, 20 µL of MTT reagent were added to each well, allowing for the cells to metabolize the reagent for 4 h. Subsequently, 100 µL of DMSO were added to dissolve the crystals formed during the metabolization. The absorbance of the resulting solutions was measured at 540 nm using a Multiskan SkyHigh spectrophotometer [5,14,53]. These absorbance values were then used to determine the CC_50_ (cytotoxic concentration 50) using OriginPro 8.5 software [50] with the Boltzmann equation (Equation (S1)). Additionally, the ratio between CC_50_ and IC_50_, the selectivity index (SI), was calculated. This value is a measure of the compound’s toxicity relative to its therapeutic efficacy. A higher ratio is better, showing a wider therapeutic window. MTT assays were performed with four technical replicates per treatment concentration (eight for controls) in each experiment. Data from each experiment were averaged, and the final results represent the mean ± SD (standard deviation) of all experiments. Similarly, randomization was applied for sample allocation, but measurements were not blinded.

### 2.7. Gene Expression Profiling

#### 2.7.1. Promastigote Treatment

*L. major*, *L. infantum*, and *L. amazonensis* promastigotes were cultured for 24 h at 26 °C in M199 1× supplemented medium. To ensure an equal parasite concentration across all experimental conditions, promastigotes were first counted in the Neubauer chamber. They were then treated with 0.25, 0.5, 1, 2.5, or 6 µM PTM concentrations below, around, and above the IC_50_ values, depending on the *Leishmania* species. A control group consisting of untreated cells supplemented with M199 1× medium was also included. Parasites were randomly assigned to treatment groups.

#### 2.7.2. RNA Extraction and Retrotranscription

RNA was extracted from both treated and untreated promastigotes using the Trizol method, which involves sequential centrifugation steps to obtain RNA dissolved in RNA-free water. Following extraction, RNA was treated with DNase and subjected to ethanol precipitation. The RNA yield was quantified using a Nanodrop NP80 spectrophotometer. cDNA synthesis was then performed using an Invitrogen^®^ kit.

#### 2.7.3. Real Time qPCR

Quantitative real-time PCR (qPCR) was conducted to analyze the expression of selected genes in response to PTM treatment, using *gapdh* as the reference gene. Gene selection was based on the functional relevance of their corresponding proteins (Table S1).

qPCR reactions were prepared in 96-well plates, each well containing 1 µL of diluted cDNA (1:10 in distilled water) and 9 µL of PCR mix (SyBR Green, primers, and distilled water). The plates were centrifuged at 1250 rpm for 2 min at 4 °C before amplification in a Bio-Rad CFX Maestro thermocycler. Real-time PCR analyses were performed in duplicate, with three technical measurements per experiment. Data from each experiment were averaged, and the final results represent the mean ± SD of all experiments.

#### 2.7.4. Statistical Analysis

Statistical analyses were performed with GraphPad Prism software (v. 9.0.1). The two-group comparisons were performed using the two-tailed unpaired t-test to determine statistical significance (***, *p* < 0.001; **, *p* < 0.01; *, *p* < 0.05).

### 2.8. Generative Artificial Intelligence (GenAI)

During the preparation of this work, Gemini AI was used to assist in generating the MATLAB script for fitting the drug release data presented in this study. After using this tool, the content was reviewed and edited as needed.

## 3. Results and Discussion

### 3.1. Production of Blow-Spun Nanofibers

Instrumental parameters such as air pressure, distance between the airbrush nozzle and the collector, PCL concentration, collector speed, and needle tip diameter affect the fiber morphology in SBS [54,55,56]. Therefore, the first step was the optimization of the conditions for the formation of the non-functionalized fibers, carried out by inspection of the blow-spun material by optical microscopy. The distance from the nozzle to the collector was set at 15 cm and 20 cm. Both distances allowed for sufficient solvent evaporation for fiber formation, but the 20 cm distance resulted in greater material loss due to material dispersion. Consequently, a 15 cm distance was selected for further experiments, although no differences in morphology were observed between the two distances. On the other hand, the variation in the pressure between 2 and 3 bar (typical range in a commercial airbrush) produced no significant differences in fiber morphology. The rotational speed of the collector, tested from 150 to 350 rpm, did affect the fiber alignment, resulting in improved alignment with higher speeds, but it had no discernible effect on the fiber width distribution and was then set at 300 rpm.

**Figure 2:**
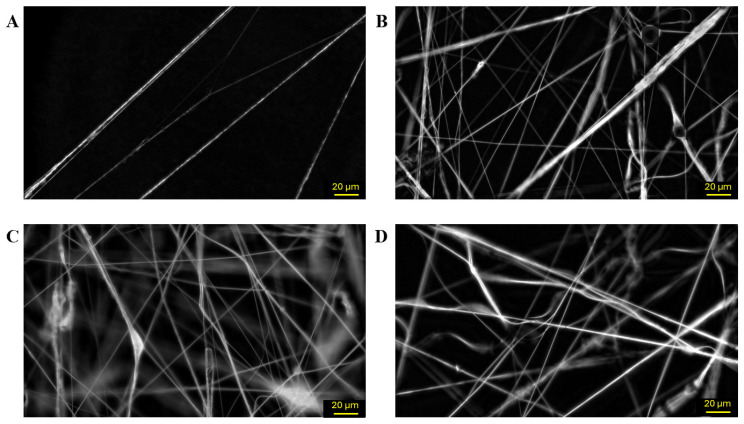
Blow-spun PCL nanofibers at different polymer concentrations as observed by polarized optical microscopy: (**A**) 4%; (**B**) 5%; (**C**) 6%; and (**D**) 7% (15 cm nozzle–collector distance, 2 bar and 0.5 mm needle tip, 400× magnification).

**Figure 3:**
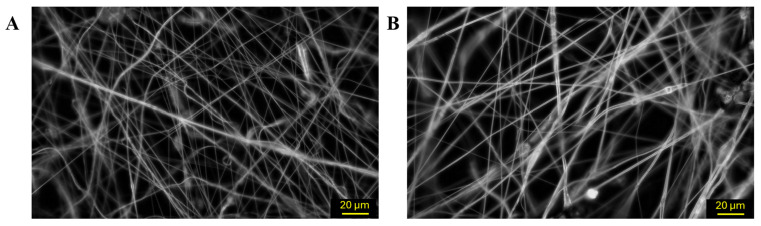
Blow-spun PCL nanofibers as observed by polarized optical microscopy at different needle tip diameters: (**A**) 0.5 mm; and (**B**) 0.3 mm (15 cm nozzle–collector distance, 2 bar and 5% PCL concentration, 400× magnification).

However, PCL concentration was a determinant parameter in the process. Concentrations below 4% (*w*/*w*) did not produce fibers, and only sparse fiber formation was observed at 4% (Figure 2A). Good structured fibers, without formation of blobs or films, were obtained between 5% and 7% (Figure 2B–D), while concentrations above 8% led to airbrush clogging due to the high viscosity of the solution. Although increasing PCL concentration influenced solution viscosity and the ease of fiber formation, it did not significantly affect fiber width. An amount of 5% PCL was identified as the optimal polymer concentration, balancing material efficiency and performance. Finally, the needle tip diameter showed only a slight influence on fiber dimensions. Optical microscopy revealed fiber widths ranging from 0.75 to 1.02 µm for the 0.3 mm tip (Figure 3A) and from 0.97 to 1.20 µm for the 0.5 mm one (Figure 3B). To further investigate the influence of the needle tip diameter, additional analyses were conducted using SEM.

**Figure 4:**
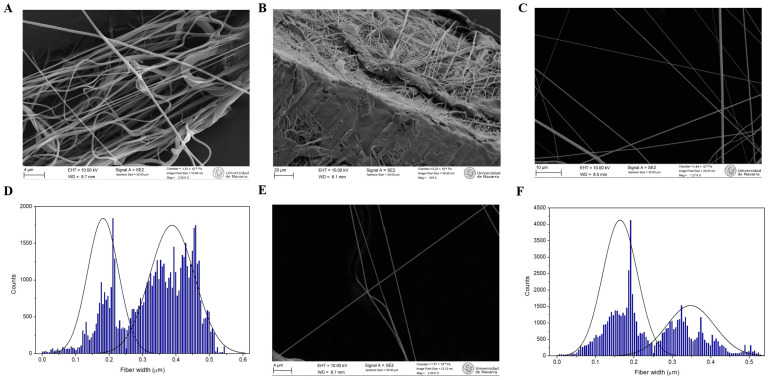
(**A**) SEM micrograph of a PCL mat obtained with the 0.5 mm diameter needle tip (2.58 K×). (**B**) SEM micrograph of a PCL mat transversal section obtained with the 0.5 mm diameter needle tip (476×). (**C**) SEM micrograph of PCL nanofibers produced by SBS with a 0.5 mm diameter needle tip (1.27 K×), and (**D**) corresponding fiber width distribution. (**E**) SEM micrograph of PCL nanofibers produced by SBS with a 0.5 mm diameter needle tip (2.30 K×), and (**F**) corresponding fiber width distribution (15 cm nozzle–collector distance, 2 bar and 5% PCL concentration in all cases).

Figure 4 and Figure S5 show selected SEM images of the nanofibers produced using both needle tip diameters, which were analyzed to determine the fibers’ width distributions. The general structure of the PCL mat obtained by SBS was examined via SEM. Images of the mat and its transversal section (Figure 4A,B) showed a uniform distribution of nanofibers throughout the entire sample, confirming the structural consistency of the fabricated material. The measurements of the fiber width distribution analyses revealed a bimodal distribution ranging from 110 to 460 nm in all samples. Fibers produced with the 0.5 mm needle tip had an average diameter of 182 ± 46 nm and 388 ± 67 nm for both distributions (Figure 4C,D), whereas those produced with the 0.3 mm one had smaller average diameter (164 ± 45 nm and 347 ± 62 nm for both distributions, Figure 4E,F). To assess whether needle tip diameter significantly influenced the fiber populations observed, two unpaired t-tests were performed, each comparing one of the two modes from the bimodal fiber width distributions corresponding to the 0.3 mm and 0.5 mm needle tips. The first test compared the lower-width distributions of both conditions, while the second evaluated the higher-width ones. In both cases, *p* < 0.0001, demonstrating statistically very significant differences between sizes.

In summary, among the processing parameters for blow-spun PCL fiber fabrication, only the needle tip diameter had a significant effect on fiber width distribution, while PCL concentration primarily influenced fiber formation feasibility (no fibers formed below 4% and clogging occurred above 8%). This is because the tip of the needle directly controls the initial diameter of the fiber at the airbrush nozzle, which critically determines the stretching of the polymer during solvent evaporation. In contrast, other instrumental parameters, such as pressure or polymer concentration, only influenced the viscosity of the solution or fiber alignment. SEM analyses showed statistically significant differences in the bimodal fiber width distributions obtained with the 0.3 mm and 0.5 mm needle tips, with the former producing thinner fibers.

### 3.2. Physicochemical Characterization of the SBS Fibers

#### 3.2.1. Physical Characterization

A uniform surface-to-weight ratio is important for a consistent drug release. Likewise, the thickness of the mat affects the diffusion pathlength. Hence, PCL-only and PCL-PTM fibers were tested for homogeneity studies by analyzing the surface-to-weight ratio and thickness of the material, as well as the drug loading for the PCL-PTM mats. Table 1 displays the surface-to-weight ratio and thickness for different PCL samples. A key consideration in the fabrication of polymeric mats by SBS is the inherent non-uniformity of fiber deposition across the collector. The polymer solution is stretched by the high-velocity air stream, resulting in a spray cone of polymeric fibers. The flux of mass thus concentrates along the central axis of the blowing nozzle and, consequently, the resulting product exhibits a central region thicker than at the peripheral edges. Along a given sample, there are only slight differences in density and thickness (Table 1), resulting in a fairly homogeneous material. However, there are variations across different samples. Both the surface-to-weight ratio and thickness directly depend on the volume of PCL solution sprayed, leading to a greater cumulative deposition of fibers on the collector area. The increased mass deposition of the polymer results in an increase in the thickness and weight of the mat, thus altering the surface-to-weight ratio. Taking the initial volume of the polymer solution into consideration may be important for controlling the final density and thickness of the mats. Therefore, for the PCL-PTM mats production, the mass of the PCL, PTM, and DCM remained constant along the different samples, resulting in more homogeneous mats in terms of thickness and density (Table 2).

The drug loading, expressed as percentage of PTM, should remain uniform across the mat to avoid dose variability. As shown in Table 2, this parameter is virtually the same in both samples (2.5 ± 0.2 and 2.6 ± 0.2%), indicating that the PTM is evenly distributed despite the poor solubility of the drug in DCM. It is worth noting that some drug is lost due to the SBS process, as the initial PTM in PCL fibers was 3.0%, resulting in an encapsulation efficacy of 83–87 ± 6%, depending on the airbrush needle tip (0.3 or 0.5 mm).

Overall, the consistent use of a fixed volume of polymer solution is key to producing homogeneous fibers. The high degree of homogeneity observed in the density, thickness, and drug loading across the samples validates the methodology used.

#### 3.2.2. Chemical Characterization via FTIR-ATR and XRD

The combined use of XRD and FTIR-ATR can shed light on the effect of the processing of the polymer, as well as on the interactions between the PCL and PTM in the fibers. The samples considered were as-received commercial PCL, a mat of PCL (5% PCL), commercial PTM (powder form), and PCL + PTM mats with low and high drug loadings (5% PCL + 3% or 20% PTM)

(A) Effect of SBS processing on the PCL structure

**Figure 5:**
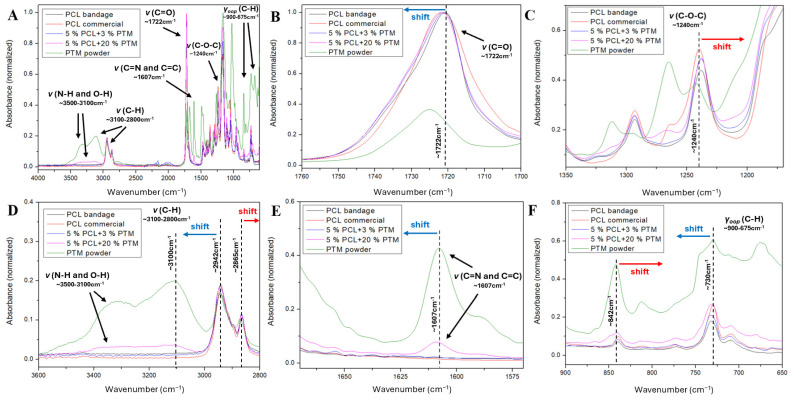
(**A**) FTIR-ATR spectra of PCL (commercial and SBS-mat), commercial PTM, and PCL + PTM (low and high drug loadings). (**B**–**F**) Expansion of different regions: (**B**) 1760–1700 cm^−1^, (**C**) 1350–1220 cm^−1^, (**D**) 3600–2800 cm^−1^, (**E**) 1680–1570 cm^−1^, (**F**) 900–650 cm^−1^.

Figure 5A shows the mid-IR spectra of all the samples under study. PCL spectra (commercial and bandage) are dominated by the strong carbonyl stretching vibration at 1722 cm^−1^ (Figure 5A,B) and the C-O-C asymmetric stretching band at 1240 cm^−1^ (Figure 5A,C), characteristic of its polyester structure [57,58,59,60,61,62]. Comparing the C=O band position (~1722 cm^−1^) between both samples (Figure 5B), a small red-shift of 1.6 cm^−1^ of the commercial PCL can be detected. On the other hand, the C-O-C vibration shifts from 1239.7 cm^−1^ in the commercial PCL to 1238.2 cm^−1^ in the mats. These small red-shifts suggest variations in the polymer’s crystalline/amorphous ratio or chain conformation with the SBS process. Moreover, the aliphatic C-H stretching modes (CH_2_ groups) of PCL provides information about the chain packing efficiency, which is sensitive to the processing of the polymer [60,61,62]. A blue shift of 1.5 cm^−1^ is observed in the C-H asymmetric stretching band of the blow-spun PCL, compared to the commercial polymer (2942.3 cm^−1^ vs. 2940.8 cm^−1^, respectively), while in the C-H symmetric stretching band a 2.6 cm^−1^ red shift occurs (2865.0 cm^−1^ vs. 2867.6 cm^−1^) (Figure 5D). These shifts suggest changes in the packing of the PCL chain when processed via SBS. In this sense, shifts in these bands could result in a decreased packing density or increased disorder.

(B) Effect of the incorporation of PTM

The C=O stretching band is a sensitive probe for analyzing polymer chain conformation and specific intermolecular interactions. While hydrogen bonding typically results in a red shift due to the weakening of the C=O bond [58,63,64], certain conditions can lead to an increase in wavenumber, particularly when competing effects, such as polymer processing and hydrogen bonding, are present [65,66]. As mentioned before, the SBS processing results in a weakening of the C=O bonds due to variations in the polymer’s crystalline/amorphous ratio. In the PCL + 3% PTM mat the drug is fully molecularly dispersed within the PCL matrix, and the C=O stretching mode blue-shifts 0.6 cm^−1^ (1721.0 cm^−1^) compared to the SBS-processed PCL (Figure 5B), suggesting the formation of stabilizing hydrogen bonds between the PCL carbonyl (hydrogen acceptor) and the O-H or N-H (hydrogen donor) of the drug. In the PCL + 20%PTM sample, the C=O band blue-shifts to 1721.6 cm^−1^, so this greater shift indicates the formation of a greater hydrogen bonding between the PTM and the PCL, although part of the PTM exits in a crystalline phase.

Upon incorporation of the drug (Figure 5C), the C-O-C asymmetric stretching bands red-shift with respect to both the commercial and blow-spun PCL samples (1239.7 and 1238.2 cm^−1^). Specifically, in the PCL + 3% PTM mat the shift is 1.8 cm^−1^ (1237.9 cm^−1^) compared to the commercial PCL, while in the PCL + 20% PTM mat the shift is 2.1 cm^−1^ (1237.6 cm^−1^). This suggests that the PTM molecules are not only interacting with the C=O group of the polymer, but are also perturbing the environment of the C-O-C ester core, further supporting the evidence formation of hydrogen bonds between the polymer and the drug.

The bands in the 3500–3200 cm^−1^ region of the PTM powder sample (Figure 5A,D) are assigned to the N-H (amidino) and O-H (isethionate) stretching modes [67]. In the PCL + 3% PTM sample, these bands are not detected, confirming the molecular dispersion of the drug in the PCL matrix at this low loading and the formation of hydrogen bonds [68]. At higher loading of PTM (20%), a low-intensity narrow N-H/O-H band appears due to the presence of the drug in crystalline form.

As discussed for the polymer alone, aliphatic C-H stretching modes of PCL reflect the polymer’s structural order [60,61,62]. The most significant result is the 2.9 cm^−1^ blue shift in the asymmetric C-H band in the PCL + 20% PTM sample (2945.2 cm^−1^) compared to the blow-spun PCL (Figure 5D), which indicates the presence of crystalline domains of PTM. Conversely, the 1.1 cm^−1^ shift in the 3% PTM sample (2943.4 cm^−1^) supports the conclusion of the dispersion of the drug at a molecular level, causing only a reduced structural disruption. The aromatic C-H stretching modes (3100 cm^−1^), exclusive of PTM [67], mark the drug’s presence (Figure 5A,D). At the 3% loading, the absence of any signal confirms that the PTM must be molecularly dispersed, while at 20%, the C-H band is detectable, indicating the presence of crystalline PTM phase, at the same wavenumber as in PTM.

The spectrum of PTM powder shows a band at 1607.5 cm^−1^, corresponding to the primary aromatic (C=C) and amidino (C=N) stretching (Figure 5A,E) [67]. At a high PTM proportion (20%), the blue shift of 1.5 cm^−1^ (1609.0 cm^−1^) indicates the disruption of the crystalline network of PTM. This band does not appear in the PCL + 3% PTM sample because the drug is molecularly dispersed and its concentration falls below the detection of the technique in the mixture. The simultaneous occurrence of the red shift in the carbonyl of PCL and blue shift in the C=C and C=N bonds in PTM strongly suggest the interaction between drug and polymer via hydrogen bonding.

The region below 900 cm^−1^ in PTM contains vibrations of the aromatic rings of PTM, specifically the out-of-plane C-H bending [69], although the analysis in this zone becomes complicated by overlap with some PCL characteristic bands (Figure 5F). In the spectrum of the PTM powder, a distinct band is observed at 842.0 cm^−1^. This signal is absent in the 3% PTM sample, but has a band at 839.6 cm^−1^ corresponding to one of the PCL, so that the drug signal is below the limit of detection of the technique. The 20% PTM-loaded sample shows a broad band at 840.3 cm^−1^, slightly red-shifted relative to the PTM one, representing the combined absorptions of both PCL and PTM. Moreover, the signal of the C-H bending at 730.1 cm^−1^ for PTM powder overlaps with the C-H rocking of CH_2_ in PCL (732.1 cm^−1^), making the analysis complicated. Both PTM-loaded bandages exhibit a slightly blue shift in this signal (1.3 cm^−1^ and 2.6 cm^−1^ for 20% and 3% drug loading, respectively), confirming the effect on the aromatic rings of the drug in the presence of the PCL matrix, and, consequently, the intermolecular interactions between the PTM and the polymer.

Finally, no bands attributable to DCM were detected in the PCL bandage or any of the PCL + PTM composite spectra, which confirms the evaporation of the DCM solvent during the SBS process.

**Figure 6:**
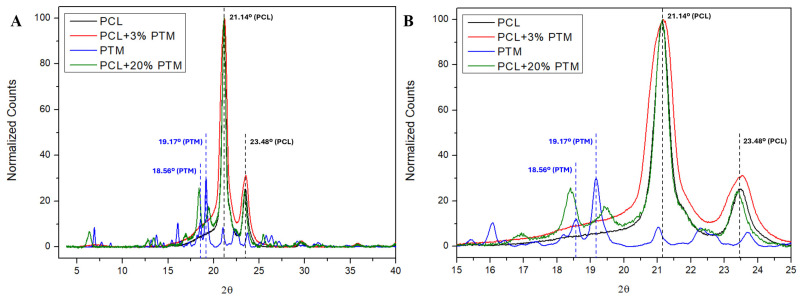
(**A**) XRD patterns for PCL (bandage), commercial PTM (powder), and PCL + PTM (low and high drug loadings). (**B**) Expansion of the 15–25° (2θ) region.

XRD was used to assess the structural changes in the PTM-loaded PCL nanofibers by comparing them to the individual PTM and PCL components. Figure 6 shows the diffractograms of the PCL (mat), commercial PTM (powder), and PCL + PTM mat at low and high drug loadings. The PTM powder exhibits its characteristic peaks, the most intense being at 18.56° and 19.17° (2θ) (Figure 6A,B), while PCL shows two intense signals at 21.14° and 23.48° (2θ) (Figure 6A,B), corresponding to the (110) and (200) planes of the semicrystalline PCL [59]. These remain unchanged upon the incorporation of PTM, as shown in Figure 6B. The PCL + 20% PTM sample displays reflections that evidence the presence of a crystalline drug phase, but at different angles (18.39° and 19.41°, 2θ) and with different relative intensities compared to the neat PTM. The polymorphism in PTM isethionate has been investigated as a function of the temperature and freeze-drying [70]. The changes observed in our diffractograms are compatible with the formation of a PTM polymorph induced by the SBS process. These results agree with the observed shifts in the PTM bands in the mid-IR (O-H and N-H stretching and C=N and C=C stretching) (Figure 5). Conversely, the 3% PTM sample does not show detectable diffractions peaks of the crystalline PTM (Figure 6A,B), a confirmation that, at low drug content, the PTM is molecularly dispersed in the polymer matrix in an amorphous state, in line with the FTIR evidence discussed above.

Taken together, the FTIR and XRD evidence provide a unified understanding of the state of PTM in PCL fibers. Both techniques show that at low loading (3%), PTM is amorphous and molecularly dispersed within the polymer, while at high loading (20%), as there is a drug excess, it remains partially crystalline, but can undergo a polymorphic transition induced by the SBS process, as evidenced by the different XRD pattern and the corresponding FTIR band shifts due to hydrogen bonding. It is worth mentioning that high drug loadings do not reflect realistic conditions for biological applications, serving instead as an experimental model to better visualize and characterize the drug–polymer interactions and structural features of the material.

### 3.3. Drug Release Kinetics and Mechanism

A 5% PCL concentration was used to evaluate the release profile of PTM from PCL nanofibers, since this concentration offers the best compromise between fiber formation and material efficiency, as established in the previous section. A pilot experiment confirmed that PTM could be reliably detected by fluorescence spectroscopy at concentrations as low as 0.1 µM under the experimental conditions. Based on this, PTM (3% relative to PCL mass) was incorporated into the polymer solution to ensure an adequate drug release for subsequent biological studies.

**Figure 7:**
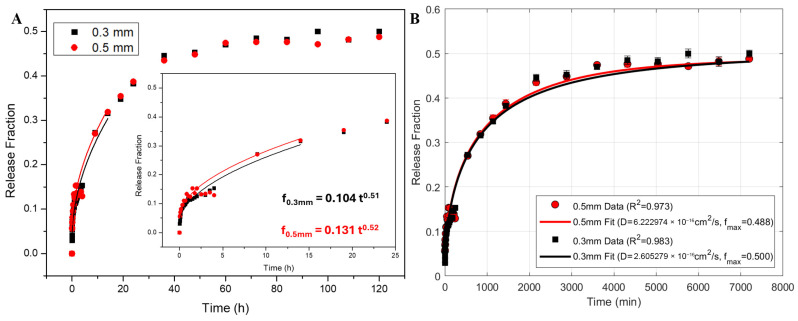
(**A**) Released PTM as a function of time for both needles fitted to KP (Equation (1)). (**B**) Released PTM as a function of time for 0.3 and 0.5 mm needle tip diameters fitted to Equation (3) using the first 20 Bessel roots.

Fiber diameter is also a key parameter in the release of drugs from fibrous materials [71]; hence, the effect of the needle tip diameter (0.3 mm vs. 0.5 mm) on the release mechanism was examined. Figure 7A shows that, irrespectively of the needle used, the release profile for both experimental set-ups is very similar, with approximately 50% of the PTM released after 60 h.

A widely used semi-empirical equation to rationalize various types of release profiles is the Korsmeyer–Peppas (KP):

(1) $$f=kt^{n}$$

where *f* is the fraction of drug released; *k* is the release rate constant; and *n* is the transport exponent associated with the release mechanism of the drug [72,73,74]. This model is commonly used in controlled release systems in which an initial rapid release stage (burst) occurs due to the adsorption of the drug on the surface of the delivery vehicle, and has been validated in various formulations, including polymeric films [6,75,76]. Equation (1) is only applied to the initial 60% of the release profile, as the model assumptions, such as constant geometry and no polymer degradation, remain unaffected. Beyond this point, additional factors like swelling or erosion may affect the release behavior, making the model less accurate [72,73,74].

The fitted kinetic parameters to the KP equation for the two needle tips are summarized in Table 3. The similar release exponent (*n*) suggests that the mechanism is the same for both fiber types. The *n* values, close to 0.5, indicate that the release is predominantly governed by Fickian diffusion [72,73,74]. The release constant is also similar, being slightly greater for the 0.5 mm needle tip diameter. In conclusion, the release kinetics of PTM from the blow-spun PCL fibers show a consistent release, unaffected by the needle tip diameter, through diffusion mechanisms.

Although the KP equation is extensively used in pharmaceutical applications, it is a semi-empirical model that defines the initial phase of the kinetic curve, where a steep increase in drug release, proportional to the square root of time for spherical and cylindrical geometries is observed [72,74]. It has been discussed that the fiber diameter distribution is a critical parameter that significantly impacts the overall drug release profile and the determination of the diffusion coefficient [71]. Therefore, they developed a mathematical model for drug release from electrospun polymeric scaffolds that incorporates the distribution of fiber diameters within the material. Unlike conventional homogeneous models that assume a uniform fiber radius, their approach accounts for the variability in fiber size observed experimentally and uses this distribution to more accurately predict drug release kinetics. By applying the Laplace transform to Fick’s second law in cylindrical coordinates, they demonstrated that fiber radius distribution significantly influences the release rate and can be used to refine the estimation of the drug diffusion coefficient within the polymer matrix [71]. This mathematical approach is much more rigorous and takes into consideration the release profile of each fiber.

The blow-spun mats consist of “infinitely” long cylinders with a given width distribution. Assuming one-dimensional radial diffusion, the Fick’s Second Law in cylindrical coordinates is

(2) $$\frac{\partial C}{\partial t}=D\left(\frac{\partial^{2}C}{{\partial r}^{2}}+\frac{1}{r}\frac{\partial C}{\partial r}\right),$$

where *r* is the radial distance from the center of the cylinder (0 ≤ *r* ≤ *R*), *C* is the concentration of the drug at the radial position *r* and time *t*, and *D* is the diffusion coefficient of the drug within the polymeric matrix. For drug release purposes, it is assumed that the concentration of the drug is uniform and equal to *C*_0_ at zero time for the entire fiber (0 ≤ *r* ≤ *R*), and that at the center (*r* = 0) there is no flux. Moreover, as the *D* of most polymers is several orders of magnitude lower than in water, once the drug reaches the surrounding medium, it is quickly dispersed [71,77].

The solution to Fick’s Second Law under the specified conditions for the cumulative fraction of drug release, f from a cylindrical matrix is an infinite series [77]:

(3) $$f=f_{max}\left(1-\sum_{n=1}^{\infty }\frac{4}{{x_{n}}^{2}}\;e^{-D\frac{{x_{n}}^{2}}{R^{2}}t}\right)$$

where *f_max_* is the maximum drug release fraction, *x_n_* are the positive roots of the Bessel functions of the first kind of zero order, and *R* is the radius of the cylinder [77]. This exact series solution (also referred to as the “unified diffusion model”) describes the entire release process from short to long times, capturing both initial, rapid release, and final sustained release [77].

We have applied a robust model for drug release considering the entire experimentally determined fiber diameter distribution of Figure 4B,D, as described by Petlin et al. (2017) [71]. We programmed a script in MATLAB implementing Equation (3) (Script S1), which considers the contribution from fibers of all diameters present in the sample and fits the experimental accumulated drug release data to the equation using a nonlinear least-squares optimization with the first 20 Bessel roots. This process minimizes the discrepancies between the observed release profiles and the model’s predictions, yielding the exact global *D* value that takes into account the complete fiber size distribution. The results are shown in Figure 7B, and the fitted parameters summarized in Table 4.

This model provides a single global diffusion coefficient for each distribution. The obtained *D* values fall within the range typically reported in the bibliography for similar drug–polymer systems, from 10^−7^ to 10^−21^ m^2^/s [78,79,80]. Although the fits with both needles produce a similar *D* values, the variation could be due to the overall porosity, tortuosity, or other microstructural features of the fiber mats. This implies that factors beyond just diameter, such as differences in packing density or internal pore structure influenced by the needle tip diameter during the blow spinning process, may be important in determining the effective diffusion coefficient, although, as discussed in the characterization section, the fibers are quite homogeneous.

Typical kinetic modeling approaches only utilize time-averaged data, neglecting the critical influence of the material’s microstructural heterogeneity. Conversely, the model here described overcomes this limitation by rigorously considering the contribution from fibers of any diameter present in the sample, offering a powerful design tool for studying drug release kinetics.

### 3.4. Activity of PTM and Nanofiber-Released PTM Against Leishmaniasis

**Figure 8:**
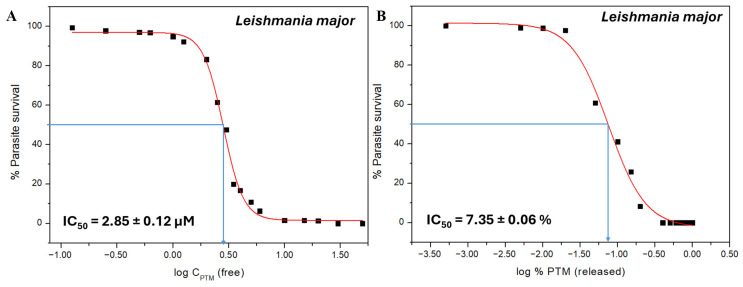
Leishmanicidal activity of PTM. Survival rates of *L. major* promastigotes treated with (**A**) free PTM (n = 2) and (**B**) nanofiber-released PTM (n = 3). IC_50_ values are provided as Mean ± SD. Experimental data are shown as black squares, the red line represents the fit obtained using the Boltzmann equation (Equation (S1)), and blue arrows indicate the graphical determination of the IC_50_ value.

The antiparasitic activity of free PTM was first evaluated against *L. major, L. infantum*, and *L. amazonensis* promastigotes. The dose–response curves obtained from the MTT assay (Figure 8 and Figure S6A,B) allow for the determination of the IC_50_ values. The IC_50_ obtained for the free PTM against *L. major* (Figure 8A) is 2.85 ± 0.12 µM, which falls within the range of the values reported in the literature (0.47–21.55 µM) [81,82,83], whereas those IC_50_ values (of free PTM) are 0.54 ± 0.04 µM for *L. infantum* and 1.18 ± 0.18 µM for *L. amazonensis* (Figure S6A,B) and very similar to those previously reported [81,82,83]. Our data indicate that *L. infantum* exhibits the highest susceptibility to PTM treatment, and *L. major* appears to be the least sensitive.

Afterwards, to determine whether the drug’s efficacy was affected by the nanofiber formulation, the antileishmanial activity of the released PTM was also assessed against *L. major*. Interestingly, the released drug follows the same pattern as the free drug, conclusively demonstrating its efficacy (Figure 8B). In fact, with 5–10% of PTM released, the survival rate of *L. major* is 40–60%, which corresponds to the 47–61% survival of parasites exposed to 2.5–3 µM (~IC_50_) of free PTM. The IC_50_ value of the nanofiber-released PTM (0–100%) is 7.35 ± 0.06% (Figure 8B). On the other hand, *Leishmania* burden is reduced to around 8% when the release of PTM from the nanofibers reaches 20%, and is comparable to the effect observed with 5–6 µM (~2-fold IC_50_) of free PTM inducing 6–11% of leishmania survival (Figure 8B).

### 3.5. Cytotoxicity of PTM Against Macrophages

The cytotoxic potential of PTM was assessed using RAW 264.7 murine macrophages. The CC_50_ of free PTM resulted in 3.37 ± 0.51 µM, and again the released drug followed the same pattern as the free drug, with a CC_50_ 31.4 ± 3.9% (Table 5, Figure S7). With 25–40% of PTM released, the macrophage survival rate was 48–54%. Interestingly, this value corresponds to 44–52% of cell viability of macrophages exposed to 3–5 µM (~CC_50_) of free PTM. When comparing the CC_50_ on macrophages (Table 5) to the IC_50_ values previously determined against different *Leishmania* species (Figure 8 and Figure S6A,B) and after calculating the SI values (Table 5), a significant variation in the therapeutic window becomes apparent. For *L. infantum* (IC_50_ = 0.54 ± 0.04 µM), the SI is 6.2, which indicates a favorable therapeutic window, suggesting that PTM could be used at concentrations several times higher than the effective parasitic dose before inducing significant host cell toxicity. For *L. major* (IC_50_ = 2.85 ± 0.12 µM) and *L. amazonensis* (IC_50_ = 1.18 ± 0.18 µM), the SI are 1.2 and 2.9, respectively. It is important to note that to successfully employ PTM as a good therapeutic option, strategies must be implemented to enhance its selectivity index and widen the therapeutic window, such as encapsulation of PTM or combination of PTM with synergistic or non-toxic agents, allowing for the use of a lower and safer dose of this drug. A higher ratio (=CC_50_/IC_50_) value (>1) is better, showing a wider therapeutic window. The current development of the PTM-loaded nanofiber mat seems to achieve a wider therapeutic window, indicating a safer, more selective drug. In fact, PTM administration through a local delivery approach aimed at overcoming some of the aforementioned therapeutic limitation by concentrating the drug at the lesion site and minimizing its cytotoxicity.

### 3.6. PTM Released from Fiber-Impaired Proliferation Pathways

To further investigate the therapeutic relevance of the nanofiber-formulated PTM, gene expression analyses were conducted to evaluate its molecular impact on parasite viability. Since drug release from polymeric systems can alter both the timing and magnitude of pharmacological effects, we assessed whether sustained PTM exposure from the nanofibers modulated critical biological pathways in *Leishmania* spp., such as the DNA replication, cell cycle, and drug resistance mechanisms. In order to illustrate the potential alteration of relevant biological functions, especially those related to *Leishmania* survival, insightful gene profiling analyses were focused on *pcna*, *top2*, *mcm4*, *prp1*, *cycA*, and *cyc6*, some critical genes belonging to the aforementioned pathways. Based on the IC_50_ values previously assessed (Figure 8A and Figure S6A,B), parasites (*L. major*, *L. infantum*, and *L. amazonensis*) were treated with different concentrations of PTM ranging from 0.5 to 6 µM and gene expression levels were accordingly evaluated.

**Figure 9:**
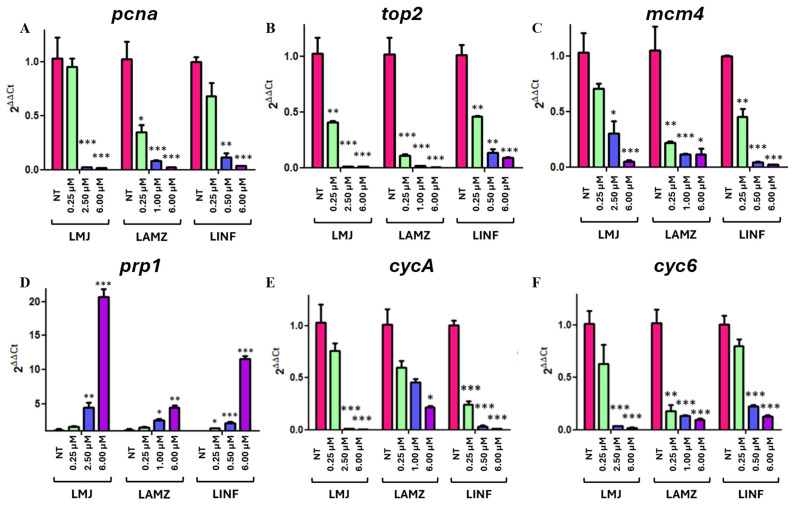
Gene expression levels after treating with 0.5–6 µM PTM. (**A**) *pcna*, (**B**) *top2*, (**C**) *mcm4*, (**D**) *prp1*, (**E**) *cycA*, and (**F**) *cyc6* in *Leishmania* spp. promastigotes (LMJ = *L. major*, LAMZ = *L. amazonensis*, LINF = *L. infantum*). All samples were analyzed in duplicate (n = 2), with three repetitions per condition. Significant differences compared to control (NT = non-treated parasites) were indicated (asterisks represent *p*-values: *, *p* < 0.05; **, *p* < 0.01; ***, *p* < 0.001).

Within the DNA replication pathway, three genes were selected: *pcna*, *top2*, and *mcm4*. *Pcna*, located in chromosome 15, spans 882 bp and encodes the proliferating cell nuclear antigen (PCNA) [84,85]. PCNA is a protein of 293 amino acids that acts as a cofactor for DNA polymerase delta, thus playing a critical role in eukaryotic DNA replication by facilitating the interaction between the mentioned polymerase and DNA [86,87]. As shown in Figure 9A, there is a significant decrease in *pcna* expression levels in the three species when increasing PTM concentrations, particularly around the IC_50_ values and at 6 µM (well above IC_50_). Indeed, in *L. major*, there is a significant reduction in the expression (of *pcna*) of 7.7% (0.25 µM PTM), 97.7% (2.50 µM = IC_50_, *p* < 0.001), and 98.3% (6.00 µM; *p* < 0.001). In *L. amazonensis*, such reduction is also relevant, and the expression decays 65.8% (0.25 µM; *p* < 0.05), 92.3% (1.00 µM = IC_50_; *p* < 0.001), and 97.8% (6.00 µM PTM; *p* < 0.001). Additionally, *L. infantum pcna* expression levels decrease by 32.3% (0.25 µM), 88.8% (0.50 µM = IC_50_; *p* < 0.01), and 96.5% (6.00 µM; *p* < 0.001). These data indicate a more marked decay in *L. major*, suggesting that PTM might have a stronger impact on *pcna* expression in this species. Since PCNA promotes DNA replication, its downregulation may significantly impair replication and reduce promastigote survival.

*Top2* is a 3.7 kb gene from chromosome 15 [88,89] that encodes topoisomerase II (TOP2), a 1236-amino acid enzyme capable of modulating DNA topology by inducing reversible breaks in DNA strands [90,91]. The expression of *top2* also significantly declines while increasing PTM concentrations in all species (Figure 9B). There is a very significant reduction in the expression levels of this gene of 60.1% (0.25 µM PTM; *p* < 0.01) and 99.0% (2.50 µM = IC_50_ and 6.00 µM, *p* < 0.001) in *L. major*. In *L. infantum* the decrease is 54.2% (0.25 µM, *p* < 0.01), 86.5% (0.50 µM = IC_50_, *p* < 0.01), and 91.3% (6.00 µM, *p* < 0.001). The most significant reductions (*p* < 0.001) are observed in *L. amazonensis,* with 89.2% (0.25 µM), 98.3% (1.00 µM = IC_50_), and 99.8% (6.00 µM), highlighting that this gene is strongly sensitive to PTM in this species. Even at low concentrations (0.25 µM), *top2* expression levels drop considerably compared to those of the control (untreated cells). Protozoan parasites, including *Leishmania*, rely heavily on DNA topoisomerases due to their complex mitochondrial DNA structure in the form of minicircles and maxicircles [19]. The PTM-induced expression changes are consistent with interference in DNA replication pathways, thus compromising the genetic integrity of parasites and inhibiting their survival [19].

*Mcm4*, a 2688 bp gene [92], encodes the minichromosome maintenance protein 4 (MCM4), which is an 895-amino acid component of the MCM helicase complex. This complex has helicase and replicative activities, being essential for several steps of DNA replication of eukaryotic cells [93,94]. The expression levels of *mcm4* also undergo a dramatic decrease with PTM concentration (Figure 9C), especially in *L. infantum* (54.75% at 0.25 µM, *p* < 0.01; 96.0% at 0.50 µM = IC_50_; and 97.7% at 6.00 µM, *p* < 0.001). In *L. major*, those levels fall by 31.6% (0.25 µM), 70.6% (2.50 µM = IC_50_; *p* < 0.05), and 95.1% (6.00 µM, *p* < 0.001). In *L. amazonensis*, there is a very significant drop at the lowest concentration (79.5% at 0.25 µM; *p* < 0.01), which then stably remain lower at higher PTM concentrations (89.2% and 89.0% at 1.00 µM (=IC_50_) and 6.00 µM, respectively). MCM4 belongs to the MCM complex and participates in DNA replication as a helicase. Its reduced expression likely disrupts MCM complex activity, hindering DNA strand separation and thereby inhibiting parasite DNA replication.

Altogether, these results reveal that changes in the expression levels of these three genes (*pcna*, *top2*, and *mcm4*) might affect DNA replication and hence reduce parasite survival. These data are consistent in *L. major*, *L. amazonensis*, and *L. infantum* treated with 0.5–6 µM PTM including those inducing the elimination of 50% of parasite burden (Figure 9A–C). Interestingly, as aforementioned (Figure 8B), when the parasites are exposed to 5–10% nanofiber-released PTM, the *Leishmania* burden is 40–60%. Therefore, in such conditions, the expression levels of *pcna, top2*, and *mcm4*, three replication-related genes, might be significantly downregulated, impairing *Leishmania* survival.

Since previous studies had reported that *prp1* expression increased through PTM treatment [95,96], we analyzed its expression levels. It is well known that *prp1* is a 5.4 kb gene within chromosome 31 [97,98], encoding a plasma membrane-associated ABC transporter. This transporter may contribute to PTM resistance and facilitate the transport of various substrates via ATP hydrolysis [99,100]. Figure 9D illustrates *prp1* expression in *L. major*, *L. amazonensis*, and *L. infantum* promastigotes when exposed to different PTM concentrations. In all three species, expression levels significantly increase compared to those of the control, and the highest expression is observed in *L. major*. At low PTM concentrations (0.25 µM), expression increases modestly: 1.6-fold in *L. major*, 1.5-fold in *L. amazonensis*, and 1.3-fold in *L. infantum*. On the contrary, at concentrations around the IC_50_ values, *prp1* expression significantly (*p* < 0.05) rise to 4.5-fold in *L. major*, 2.6-fold in *L. amazonensis*, and 2.1-fold in *L. infantum*. At 6.00 µM, such levels remain higher (20.7-fold in *L. major*, 4.5-fold in *L. amazonensis*, and 11.6-fold in *L. infantum)* (Figure 9D). These results support the idea that *prp1* plays a role in PTM resistance, especially in *L. major*. As previously discussed, PRP1 is an ABC transporter that confers pentamidine resistance in *Leishmania* spp. [95,101]. It has been proposed that PRP1 facilitates the intracellular sequestration of PTM into organelles, followed by its removal from the cell via exocytosis [95,101]. Our results confirm that the expression of *prp1* increased in response to nanofiber-released PTM treatment, suggesting the induction of strategies allowing for drug resistance and parasite survival. Currently, the precise mechanism is not yet fully understood, and further studies are needed, such as co-treatment with an efflux inhibitor followed by measurement of intracellular PTM levels.

Given the potential impact of PTM on genes regulating the parasite cell cycle, the expression levels of two cyclins, *cycA* and *cyc6*, were assessed. Cyclins are a group of proteins capable of regulating cell cycle and division [102,103]. *CycA* is a 930 bp gene located in chromosome 25 [104,105], which encodes the 309-amino acid cyclin A protein (CYCA). *Cyc6* is a 912 bp gene located in chromosome 32 [106,107] encoding cyclin 6 (CYC6), a protein of 303 amino acids [108,109]. Figure 9E,F shows the lowering expression levels of *cycA* and *cyc6* after increasing PTM exposure in *L. major*, *L. amazonensis*, and *L. infantum* species. Regarding *cycA* (Figure 9E), its expression decreases with PTM concentrations in the three species. The most substantial reduction occurs in *L. major*, with expression dropping by 26.0% (0.25 µM), 99.0% (2.50 µM = IC_50_, *p* < 0.01), and 99.6% (6.00 µM, *p* < 0.01). In *L. infantum* the expression decreases very significantly (*p* < 0.01) and rapidly (75.5% at 0.25 µM, 96.7% at 0.50 µM = IC_50_, and 99.0% at 6.00 µM). In contrast, *L. amazonensis* shows a more moderate reduction: 40.8% (0.25 µM), 55.2% (1.00 µM = IC_50_), and 78.5% (6.00 µM, *p* < 0.05).

On the other hand, the expression of *cyc6* (Figure 9F) also declines through PTM treatment. The highest reduction was detected in *L. major* (38.0% at 0.25 µM, 96.3% at 2.50 µM (= IC_50_; *p* < 0.001) and 98.1% at 6.00 µM; *p* < 0.001). *L. infantum* shows a more gradual decrease, 20.8% (0.25 µM), 77.6% (0.50 µM = IC_50_), and 87.0% (6.00 µM), while *L. amazonensis* displays a stronger response at lower concentrations: 82.2% (0.25 µM), 87.1% (1.00 µM = IC_50_; *p* < 0.001), and 90.3% (6.00 µM, *p* < 0.001). While *cycA* expression is more significantly affected in *L. major* and *L. infantum*, *cyc6* is more consistently downregulated in all three species, especially in *L. amazonensis*. Since cyclins are required for the activation of Cyclin-Dependent Kinases (CDKs), their downregulation likely impairs CDK function and thereby inhibits parasite division. Therefore, the concentrations of PTM or nanofiber-released PTM able to reduce parasite burden at 50% may also induce a very significant decrease in *cycA* and *cyc6* expression levels, impairing leishmania cell cycle and division.

## 4. Conclusions

We have described the successful production by SBS for the first time for sprayable mats for the treatment of CL via the functionalization with PTM of PCL submicrometric fibers. The initial optimization of the SBS process demonstrated the influence of the needle tip diameter of the airbrush on the resulting fiber morphology, while the PCL concentration only affected the solution’s viscosity and fiber formation efficiency. Both physical (density and thickness) parameters and drug loading were consistent across samples, validating the methodology for the production of PTM-loaded PCL fibers suitable for topical administration. The blow-spun PCL nanofiber matrix facilitated a sustained release of approximately 50% of the PTM after 60 h. Initial kinetic analysis using the KP model indicated a Fickian diffusion mechanism, independent of the needle employed. A rigorous mathematical release model, which accounts for the complete fiber’s diameter distribution, confirmed the diffusion as the predominant release mechanism. FTIR and XRD studies confirmed that the PTM is in amorphous state, molecularly dispersed, at low proportions of the drug, while at high loading there is a residual crystallinity due to polymorphic transitions induced by SBS, confirmed by the FTIR spectra. The PTM released from the PCL nanofibers exhibited significant in vitro antileishmanial activity against *Leishmania spp*., highlighting the potential of this drug delivery system for the treatment of the disease. Although PTM exhibited inherent cytotoxicity, highlighting the need to improve its selectivity and therapeutic window for safe use, embedding in PCL nanofibers aims to provide a localized delivery approach that may concentrate the drug at the lesion site, enhancing therapeutic efficacy while minimizing systemic toxicity, and thus addressing a key limitation of PTM as a good (safer and more selective) option for treatment. Mechanistically, the released PTM may disrupt key parasitic processes by downregulating several critical genes (*pcna*, *top2*, *mcm4*) involved in DNA replication and integrity, and its leishmanicidal activity is consistent with the inhibition of cell division through the impairment of *cycA* and *cyc6* expression levels, thereby contributing globally to its significant antileishmanial effect. Notably, the observed upregulation of the *prp1* gene suggests an active response mechanism by the parasite to overcome the leishmanicidal activity of PTM, likely involving an attempt to enhance drug efflux and reduce its intracellular concentration. Overall, this sprayable mat represents a novel and effective strategy for in situ drug delivery on skin wounds caused by *Leishmania* spp., and potentially for other cutaneous infections. To further support its therapeutic relevance, in vivo studies will be performed to evaluate the efficacy, biocompatibility, and translational potential of this delivery formulation, as well as to validate molecules and pathways that may contribute to the cidal activity of the drug and to better understand the mechanisms of action involved.

## Figures and Tables

**Table 1:** Summary of the surface/weight ratios and thickness of PCL mats obtained at different polymer concentrations (5–7%), nozzle-collector distance (15, 20 cm), collector speed (150, 250, 300, and 350 rpm), and needle tip diameter (0.3 and 0.5 mm). All samples were analyzed in triplicate (n = 3); results expressed as Mean ± SD (standard deviation).

Sample	Surface-Weight Ratio (cm^2^/mg)	Thickness at the Center (mm)	Thickness at the Edges (mm)
5% PCL, 15 cm, 150 rpm, 0.5 mm	0.316 ± 0.008	0.19 ± 0.01	0.11 ± 0.01
5% PCL, 15 cm, 250 rpm, 0.5 mm	0.273 ± 0.011	0.27 ± 0.01	0.15 ± 0.02
5% PCL, 15 cm, 350 rpm, 0.5 mm	0.193 ± 0.008	0.41 ± 0.00	0.22 ± 0.02
5% PCL, 15 cm, 300 rpm, 0.3 mm	0.159 ± 0.004	0.56 ± 0.03	0.15 ± 0.01
5% PCL, 20 cm, 300 rpm, 0.5 mm	0.184 ± 0.009	0.45 ± 0.04	0.13 ± 0.01
6% PCL, 15 cm, 150 rpm, 0.5 mm	0.354 ± 0.004	0.19 ± 0.02	0.11 ± 0.01
6% PCL, 15 cm, 250 rpm, 0.5 mm	0.410 ± 0.016	0.19 ± 0.01	0.10 ± 0.02
6% PCL, 15 cm, 350 rpm, 0.5 mm	0.250 ± 0.004	0.29 ± 0.02	0.11 ± 0.01
6% PCL, 15 cm, 300 rpm, 0.3 mm	0.202 ± 0.007	0.43 ± 0.01	0.11 ± 0.01
6% PCL, 20 cm, 300 rpm, 0.5 mm	0.358 ± 0.012	0.24 ± 0.02	0.11 ± 0.01
7% PCL, 15 cm, 300 rpm, 0.3 mm	0.207 ± 0.004	0.43 ± 0.01	0.11 ± 0.01

**Table 2:** Summary of surface/weight ratios, thickness, drug loading, and encapsulation efficacy of PCL/PTM mats obtained with different needle tip diameters (0.3 and 0.5 mm). All samples were analyzed in triplicate (n = 3); results expressed as Mean ± SD.

Sample	Surface-Weight Ratio (cm^2^/mg)	Thickness at the Center (mm)	Thickness at the Edges (mm)	Drug Loading (%)	Encapsulation Efficacy (%)
5% PCL, 3% PTM, 0.5 mm	0.17 ± 0.02	0.49 ± 0.04	0.23 ± 0.03	2.5 ± 0.2	83 ± 6
5% PCL, 3% PTM, 0.3 mm	0.13 ± 0.02	0.51 ± 0.09	0.22 ± 0.02	2.6 ± 0.2	87 ± 6

**Table 3:** Summary of the parameters of Equation (1) (results expressed as (Mean ± SD).

Needle	0.3 mm	0.5 mm
*k*	0.104 ± 0.004	0.131 ± 0.003
*n*	0.51 ± 0.04	0.52 ± 0.08

**Table 4:** Summary of fitted parameters for Equation (3) applied to PTM release from blow-spun PCL fibers.

Needle Tip Diameter	0.3 mm	0.5 mm
*f_max_*	0.500	0.488
*D*, Mean ± SD (m^2^/s)	(2.61 ± 0.35) × 10^−20^	(6.22 ± 1.01) × 10^−20^

**Table 5:** Summary of the IC_50_, CC_50_ values, and their ratio for PTM free and released samples. For cytotoxicity assays, free PTM samples were analyzed in quadruplicate (n = 4), while released PTM samples were analyzed in triplicate (n = 3).

Sample	IC_50_ (Mean ± SD)	CC_50_ (Mean ± SD)	Ratio (CC_50_/IC_50_)
Free PTM	2.85 ± 0.12 µM	3.37 ± 0.51 µM	1.2
Released PTM	7.35 ± 0.06%	31.40 ± 3.90%	4.3

## Data Availability

The raw and processed data supporting the findings of this study are available from the corresponding authors upon reasonable request.

## Supplementary Materials

The following supporting information can be downloaded at https://www.mdpi.com/article/10.3390/polym18020170/s1. Figure S1: Experimental set-up for fiber production via SBS. The reservoir contains a PCL-PTM solution; Figure S2: Comparison of the two needle tips used in the airbrush (diameters 0.3 mm and 0.5 mm); Figure S3: Bandage of fibers obtained by SBS; Figure S4: Selected regions of the mats used for the homogeneity tests; Figure S5: SEM micrographs of PCL microfibers produced by SBS (5% PCL, 15 cm distance nozzle-collector, 2 bar, 0.5 mm tip needle); Figure S6: Percentage of survival of (A) *L. infantum* and (B) *L. amazonensis* promastigotes treated with PTM; Figure S7: Percentage of survival of macrophages treated with (A) free PTM and (B) nanofibers-released PTM; Video S1: Nanofiber production via SBS. The footage shows the continuous deposition of fibers on the rotating cylindrical collector (5% PCL, 15 cm distance nozzle-collector, 2 bar, 0.5 mm tip needle); Equation (S1): Boltzmann equation; Table S1: Summary of the selected genes for the qPCR and the corresponding functions and primers (Fw = forward primer; Rv = reverse primer). All primers are presented in the 5’ → 3’ direction; Script S1: MATLAB script for fitting the polydisperse Fickian model (Equation (3)).
