# Supplementary material for: Pentamidine-Functionalized Polycaprolactone Nanofibers Produced by Solution Blow Spinning for Controlled Release in Cutaneous Leishmaniasis Treatment
Source: Polymers (Basel). 2026 Jan 8;18(2):170. doi: 10.3390/polym18020170 (PMC12846011; doi:10.3390/polym18020170)
Supplement: Supplementary file 1 [file polymers-18-00170-s001.zip › Supporting information-final version-GG-PN-NG.pdf]

Pentamidine-Functionalized Polycaprolactone Nanofibers Produced by Solution Blow Spinning for Controlled Release in Cutaneous Leishmaniasis Treatment

Nerea Guembe-Michel [1], Paul Nguewa [2,*] and Gustavo González-Gaitano [1,*]

[1] Department of Chemistry, School of Science, University of Navarra, 31080 Pamplona, Spain; nguembe.1@alumni.unav.es (N.G.-M.); gaitano@unav.es (G.G.-G.).

[2] Department of Microbiology and Parasitology, Navarra Institute for Health Research (IdisNA), University of Navarra, 31080 Pamplona, Spain; panguewa@unav.es (P.N.).

* Correspondence: panguewa@unav.es; gaitano@unav.es

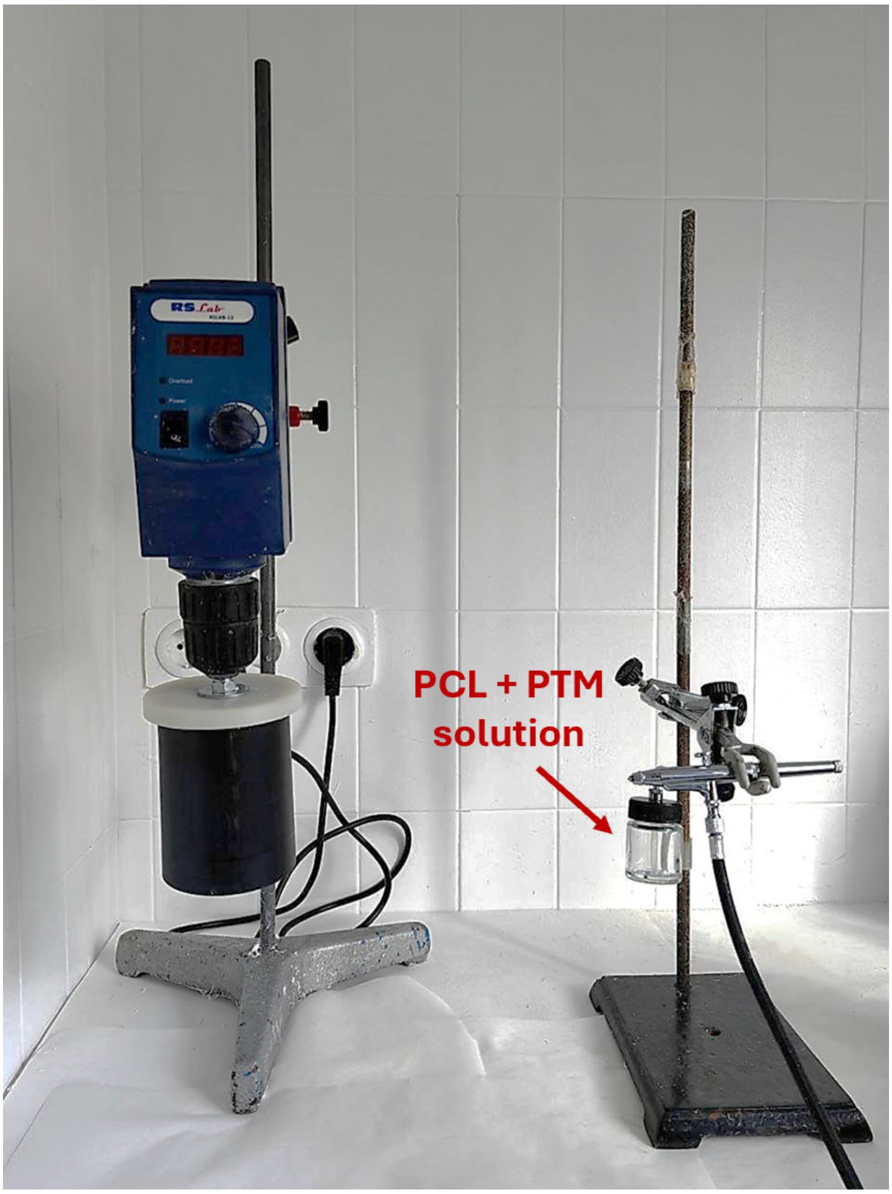

Figure S1. Experimental set-up for fiber production via SBS. The reservoir contains a PCL-PTM solution.

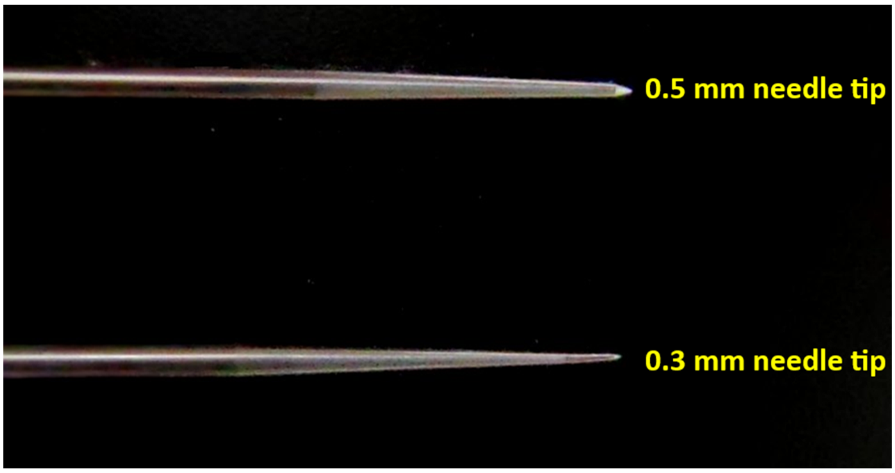

Figure S2. Comparison of the two needle tips used in the airbrush (diameters 0.3 mm and 0.5 mm).

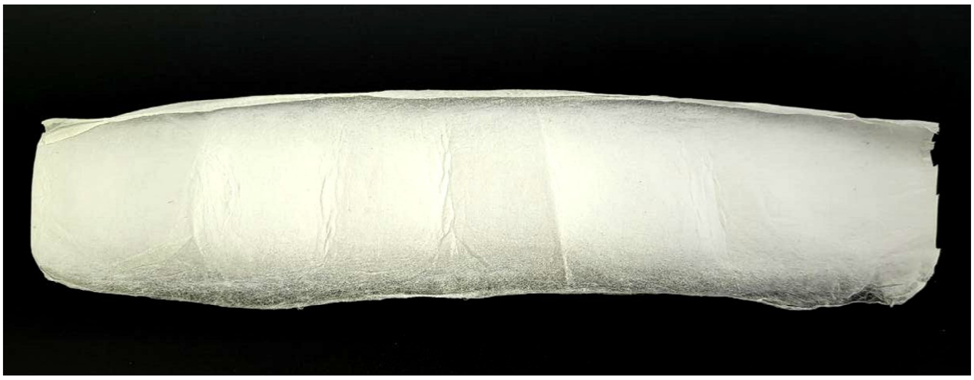

Figure S3. Bandage of fibers obtained by SBS.

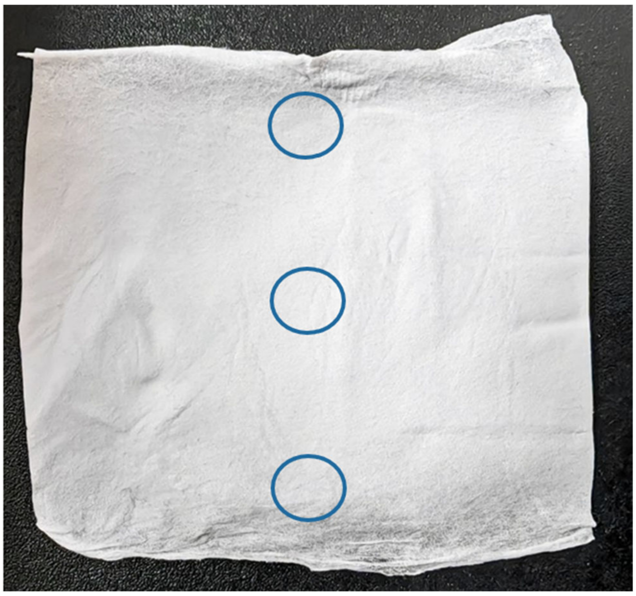

Figure S4. Selected regions of the mats used for the homogeneity tests.

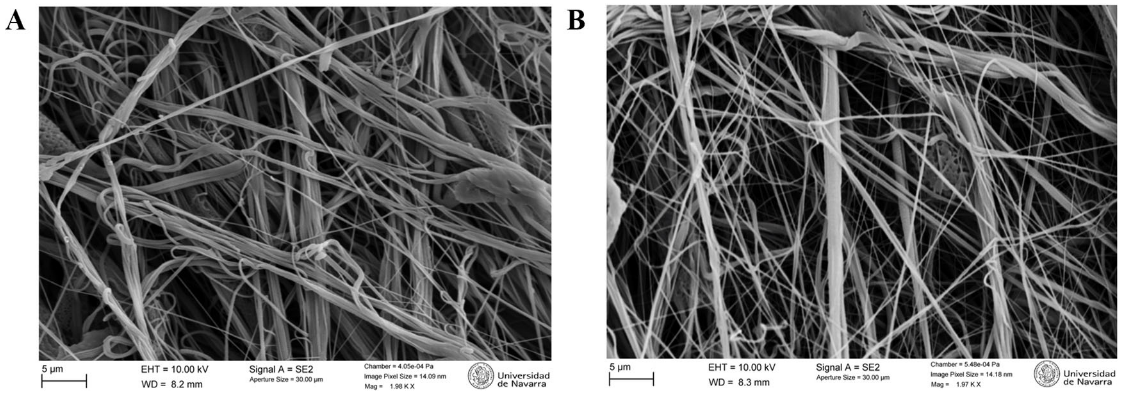

Figure S5. SEM micrographs of PCL microfibers produced by SBS (5% PCL, 15 cm distance nozzle-collector, 2 bar, 0.5 mm tip needle).

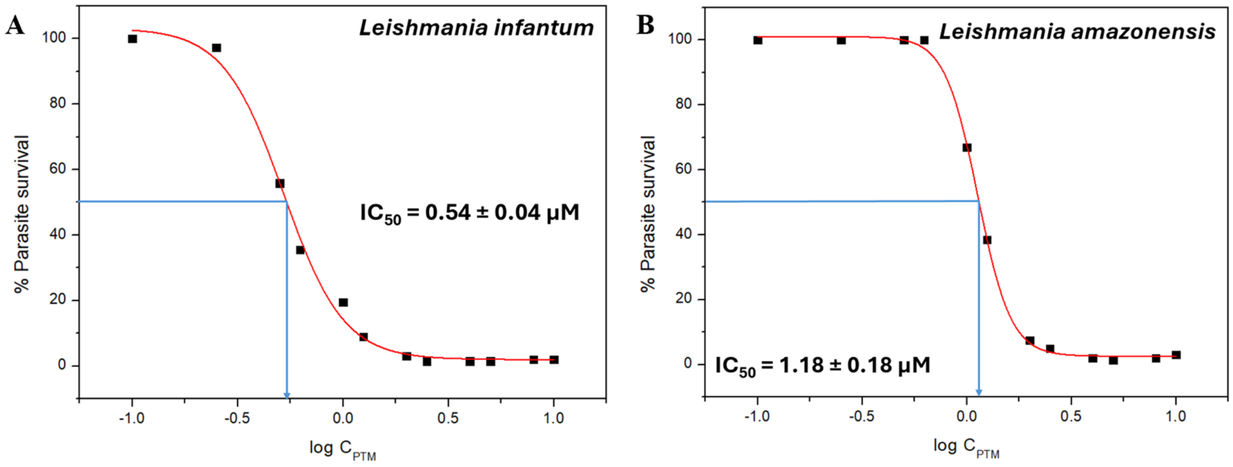

Figure S6. Percentage of survival of A) *L. infantum* and B) *L. amazonensis* promastigotes treated with PTM.

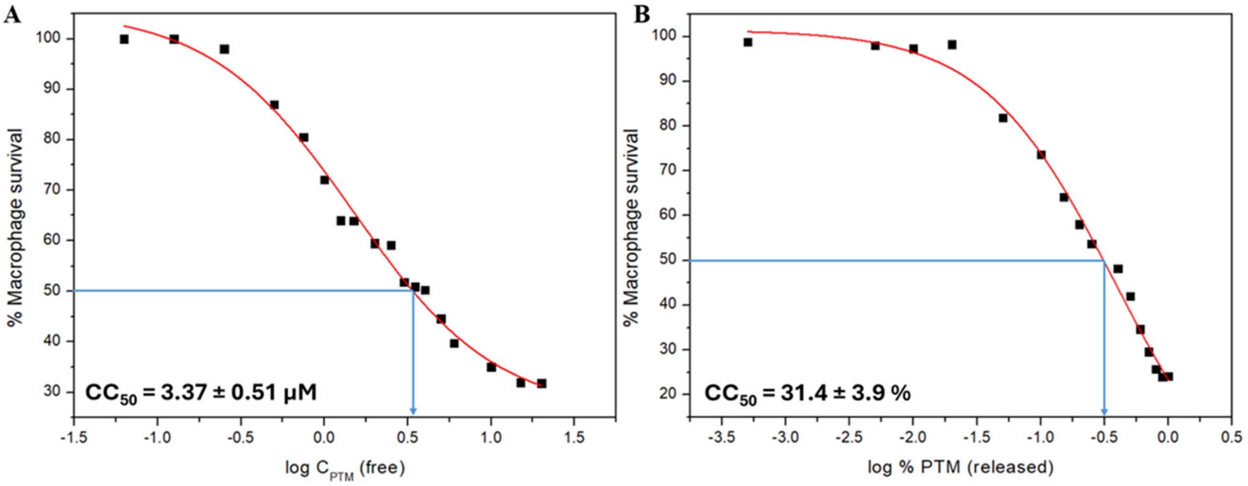

Figure S7. Percentage of survival of macrophages treated with A) free PTM and B) nanofibers-released PTM.

Video 1.mp4

Video S1. Nanofiber production via SBS. The footage shows the continuous deposition of fibers on the rotating cylindrical collector (5% PCL, 15 cm distance nozzle-collector, 2 bar, 0.5 mm tip needle).

Boltzmann equation (**Eq. S1**) implemented in OriginPro software:

$$y = A_2 + \frac{A_1 - A_2}{1 + e^{(x - x_0)/dx}}, \qquad \text{(S1)}$$

where A_1 is the initial value (upper pleateau), A_2 the final value (lower plateau), x_0 the inflection point (midpoint) and dx the slope factor, related to the steepness of the curve.

Table S1. Summary of the selected genes for the qPCR and the corresponding functions and primers (Fw = forward primer; Rv = reverse primer). All primers are presented in the 5' → 3' direction.

**Protein (Function)**	**Gene(s)**	**Primers (5' → 3')**
GAPDH (Glycolytic enzyme)	***gapdh*** *(reference gene)*	Fw: ACCACCATCCACTCCTACA
		Rv: CGTGCTCGGGATGATGTTTA
Cyclin A<br>Cyclin 6<br>(Cell cycle and division proteins)	***cycA***	Fw: CCCCAACACCGCTGACTAAT
		Rv: TCCGACTGGCGGTCTATGTA
	cyc6	Fw: AGTACCCTGCACGCCTACTA
		Rv: TTGTTGTTGGCGCAGGAAAG
Pentamidine Resistance Protein 1, PRP1<br>(PTM-resistant functions)	***prp1***	Fw: CTCATGCGTCAGTGCAAGTG
		Rv: AAACAACGGGCAAAAAGCGA
Proliferating Cell Nuclear Antigen, PCNA<br>Topoisomerase II, TOP2<br>Minichromosome Maintenance Protein 4, MCM4<br>(DNA replication activity)	***pcna***	Fw: AGATGGACTACCGCAGCA
		Rv: CTCTGATTTCACCTCCGACTTG
	top2	Fw: AGTATAAGAAGCTCACCCCG
		Rv: GTTGTTGATGTTGTCTGCCG
	mcm4	Fw: CGAGTTCGACAAGATGAACG
		Rv: ATTCCACTGTGAGTCCTTCG

Script S1. MATLAB script for fitting the polydisperse Fickian model (**Eq. 3**).

```
%% Code for fitting the 2nd Fick's Law to drug delivery data
clear all; close all; clc;

%% ========================================================================
% 1. Global Configuration and Definition of Conditions
%% ========================================================================
excelFileName_diameters = 'diametros_fibras.xlsx'; % Upload fiber data
excelFileName_release = 'Cinéticas PCL+PTM.xlsx'; % Upload kinetic data
dataRange_diameters = 'A:A';
dataRange_release = 'A:C';

conditions_internal = {'mm05','mm03'};
conditions_display = {'0.5mm','0.3mm'};
sheetMap_diameters = containers.Map(conditions_internal,{'05mm','03mm'});
sheetMap_release   = containers.Map(conditions_internal,{'0.5mm','0.3mm'});
display_name_map   = containers.Map(conditions_internal,conditions_display);

time_conversion_factor_min_to_s = 60;
conversion_factor_um_to_cm = 1e-4;

num_bins = 130;
num_bessel_roots = 20;

% Compute Bessel function roots
x_n = zeros(1,num_bessel_roots);
```

```
for n = 1:num_bessel_roots
    if n==1
        x_n(n) = fzero(@(x) besselj(0,x),2.4);
    else
        x_n(n) = fzero(@(x) besselj(0,x),(n-1+0.75)*pi);
    end
end
fprintf('First %d Bessel roots (x_n) calculated.\n', num_bessel_roots);

results_data = struct();

options = optimset('Display','off','MaxFunEvals',10000,'MaxIter',1000,'TolFun',1e-
12,'TolX',1e-12);

% Plot settings for each condition
plot_settings = containers.Map();
plot_settings('mm05') = struct('color','r','line_style','-','marker_style','o');
plot_settings('mm03') = struct('color','k','line_style','-','marker_style','s');

% Initial guesses for diffusion coefficients
initial_guesses_D_ranges = containers.Map();
initial_guesses_D_ranges('mm05') = logspace(log10(1e-17),log10(1e-15),30);
initial_guesses_D_ranges('mm03') = logspace(log10(1e-17),log10(1e-15),30);

lb_D = 1e-18; ub_D = 1e-6; % Lower and upper bounds for D

%% ============================================================================
% 2. Loop over each condition
%% ============================================================================
for k = 1:length(conditions_internal)
    current_condition_internal_name = conditions_internal{k};
    current_display_name = display_name_map(current_condition_internal_name);

    fprintf('\n--- Processing condition: %s ---\n', current_display_name);

    %% a. Load and prepare fiber diameter data
    try
        diameters_um_raw = xlsread(excelFileName_diameters,
sheetMap_diameters(current_condition_internal_name), dataRange_diameters);
    catch ME
        fprintf(2,'ERROR loading diameters for %s. Message:
%s\n',current_display_name,ME.message);
        continue;
    end
    diameters_um_raw = diameters_um_raw(isfinite(diameters_um_raw) &
diameters_um_raw>0);
    if isempty(diameters_um_raw), continue; end

    [counts,edges] = histcounts(diameters_um_raw,num_bins);
    valid_bins_idx = counts>0;
    counts = counts(valid_bins_idx);
    first_valid_edge_idx = find(valid_bins_idx,1,'first');
    last_valid_edge_idx  = find(valid_bins_idx,1,'last')+1;
    edges_valid = edges(first_valid_edge_idx:last_valid_edge_idx);
    diameters_um = (edges_valid(1:end-1)+edges_valid(2:end))/2;
    p_values = counts/sum(counts);
    R_values = (diameters_um/2)*conversion_factor_um_to_cm;

    %% b. Load experimental drug release data
    try
        releaseData_raw = xlsread(excelFileName_release,
sheetMap_release(current_condition_internal_name), dataRange_release);
        time_exp_raw_s = releaseData_raw(:,1)*time_conversion_factor_min_to_s;
        release_exp_raw = releaseData_raw(:,2);
        release_error_raw = zeros(size(release_exp_raw));
        if size(releaseData_raw,2)>=3
```

```
            release_error_raw = releaseData_raw(:,3);

release_error_raw(~isfinite(release_error_raw)|release_error_raw<=0)=0.001;
        else
            release_error_raw = 0.001*ones(size(release_exp_raw));
        end
        Q0_at_t0_from_data = 0;
        t0_idx = find(time_exp_raw_s==0,1);
        if ~isempty(t0_idx), Q0_at_t0_from_data = release_exp_raw(t0_idx); end
        idx_positive_time = time_exp_raw_s>0;
        time_exp_s = time_exp_raw_s(idx_positive_time);
        release_exp = release_exp_raw(idx_positive_time);
        release_error = release_error_raw(idx_positive_time);
    catch ME
        fprintf(2,'ERROR loading release data for %s. Message:
%s\n',current_display_name,ME.message);
        continue;
    end
    M_total_at_steady_state = max(release_exp_raw);
    if M_total_at_steady_state<=0, continue; end

    %% c. Fit in log(D)
    logD_model = @(logD,t)
local_polydisperse_fickian_model(10.^logD,t,R_values,p_values,x_n,M_total_at_steady_s
tate);
    initial_logD_guesses =
log10(initial_guesses_D_ranges(current_condition_internal_name));

    best_R_squared = -inf; best_fitted_logD = NaN;
    best_residual = []; best_exitflag = NaN; best_output = [];

    for i = 1:length(initial_logD_guesses)
        current_initial_logD = initial_logD_guesses(i);
        [fitted_logD_trial,resnorm_trial,residual_trial,exitflag_trial,output_trial]
= ...

lsqcurvefit(logD_model,current_initial_logD,time_exp_s,release_exp,log10(lb_D),log10(
ub_D),options);

        SS_tot = sum((release_exp - mean(release_exp)).^2);
        R_squared_trial = 1 - sum(residual_trial.^2)/SS_tot;

        if R_squared_trial>best_R_squared
            best_R_squared = R_squared_trial;
            best_fitted_logD = fitted_logD_trial;
            best_residual = residual_trial;
            best_exitflag = exitflag_trial;
            best_output = output_trial;
        end
    end

    %% d. Compute 95% confidence interval in log(D) and convert to D
    try
        [~,~,~,~,~,~,J] =
lsqcurvefit(logD_model,best_fitted_logD,time_exp_s,release_exp,log10(lb_D),log10(ub_D
),options);
        CI_logD = nlparci(best_fitted_logD,best_residual,'jacobian',J);
        CI_D = 10.^CI_logD;
    catch
        CI_D = [NaN NaN];
    end
    best_fitted_D = 10.^best_fitted_logD;

    fprintf('--- Best Fitted Parameters for %s ---\n',current_display_name);
    fprintf('D: %.4e cm^2/s, 95%% CI: [%.4e, %.4e]\n',best_fitted_D,CI_D(1),CI_D(2));
    fprintf('R-squared: %.4f\n',best_R_squared);
```

```
    results_data.(current_condition_internal_name) = struct(...

'time_exp_s',time_exp_s,'release_exp',release_exp,'release_error',release_error,...
        'Q0_at_t0_from_data',Q0_at_t0_from_data,'fitted_D',best_fitted_D,...

'M_total_at_steady_state',M_total_at_steady_state,'R_squared',best_R_squared,...

'R_values',R_values,'p_values',p_values,'display_name',current_display_name,'CI_D',CI
_D);
end

%% ============================================================================
% 3. Visualization
%% ============================================================================
figure('Name','Drug Release Fit'); hold on;

for k = 1:length(conditions_internal)
    cond_name = conditions_internal{k};
    data = results_data.(cond_name);
    settings = plot_settings(cond_name);
    color = settings.color; line_style = settings.line_style; marker_style =
settings.marker_style;

    % Experimental points
    plot(data.time_exp_s/time_conversion_factor_min_to_s, data.release_exp,
marker_style, ...
        'MarkerSize', 7, 'MarkerFaceColor', color, 'MarkerEdgeColor', 'k', ...
        'DisplayName', [data.display_name ' Data (R^2='
num2str(data.R_squared,'%.3f') ')']);

    % Initial point Q0 if exists
    if data.Q0_at_t0_from_data > 0
        plot(0, data.Q0_at_t0_from_data, 'o', 'MarkerSize', 7, 'MarkerFaceColor',
color*0.8, ...
             'MarkerEdgeColor', 'k', 'HandleVisibility', 'off');
    end

    % Fitted curve
    time_fit_s = linspace(min(data.time_exp_s), max(data.time_exp_s), 500);
    f_fit = local_polydisperse_fickian_model(data.fitted_D, time_fit_s,
data.R_values, data.p_values, x_n, data.M_total_at_steady_state);
    plot(time_fit_s/time_conversion_factor_min_to_s, f_fit, line_style, 'LineWidth',
2, 'Color', color, ...
    'DisplayName', [data.display_name ' Fit (D=' num2str(data.fitted_D,'%e') '
cm^2/s, f_{max}=' num2str(data.M_total_at_steady_state,'%.3f') ')']);

    % Error bars
    if ~isempty(data.release_error)
        errorbar(data.time_exp_s/time_conversion_factor_min_to_s, data.release_exp,
data.release_error, [color '.'], 'HandleVisibility','off');
    end
end

xlabel('Time (min)');
ylabel('Release Fraction');
legend('show','Location','southeast');
grid on; box on;
hold off;

%% ============================================================================
% 4. Local Function
%% ============================================================================
function model_output =
local_polydisperse_fickian_model(D_param,t_input,R_vals,p_vals,x_n_roots,M_total_stea
dy_state)
```

```
    t_col_vector = t_input(:);
    num_time_points = length(t_col_vector);
    num_bins = length(R_vals);
    num_bessel_roots = length(x_n_roots);

    x_n_sq = reshape(x_n_roots.^2,1,num_bessel_roots,1);
    R_sq   = reshape(R_vals(:).^2,num_bins,1,1);
    t_reshaped = reshape(t_col_vector,1,1,num_time_points);

    exp_arg = -D_param*(x_n_sq./R_sq).*t_reshaped;
    exp_matrix = exp(exp_arg);
    term_n_matrix = (4./x_n_sq).*exp_matrix;
    series_sum = sum(term_n_matrix,2);
    series_sum_2D = squeeze(series_sum);
    fickian_per_bin = 1 - series_sum_2D;
    weighted_fickian = p_vals(:).*fickian_per_bin;
    total_fickian = sum(weighted_fickian,1);
    model_output = M_total_steady_state*total_fickian(:);
end
```
